# Assessment of atherosclerotic plaques in the rabbit abdominal aorta with interleukin-8 monoclonal antibody-targeted ultrasound microbubbles

**DOI:** 10.1007/s11033-012-2382-5

**Published:** 2013-01-05

**Authors:** Yongping Lu, Jia Wei, Qinghua Shao, Yueyue Tang, Yanling Huang, Huali Zhang, Wei Yang, Zhi Jing

**Affiliations:** 1 Department of Ultrasound, The Fourth Affiliated Hospital of Kunming Medical University, The Second People’s Hospital of Yunnan Province, Qingnian Road 176, Kunming, 650021 China; 2 The Fourth Affiliated Hospital of Kunming Medical University, The Second People’s Hospital of Yunnan Province, Kunming, 650021 China

**Keywords:** Contrast-enhanced sonography, IL-8, Targeted microbubbles, Atherosclerotic plaque

## Abstract

In this study, we aimed to prepare a neovascularization-relevant inflammatory cytokine-targeted ultrasound contrast agent and apply it in the ultrasound imaging of atherosclerotic plaque. An interleukin-8 (IL-8) monoclonal antibody was conjugated to SonoVue microbubbles using the *N*-succinimidyl-3-(2-pyridyldithio)propionate cross-linking method. Then, a prepared IL-8-targeted contrast agent was used for contrast-enhanced ultrasound (CEU) to detect rabbit abdominal aorta atherosclerotic plaque and to investigate the imaging characteristics of atherosclerotic plaque with the contrast agent. We found that an IL-8 monoclonal antibody can be successfully coupled to SonoVue microbubbles with stable biological characteristics. CEU with this IL-8-targeted contrast agent can increase the atherosclerotic plaque detection sensitivity, with stronger echo, so that three more plaques were detected compared with using non-targeted SonoVue microbubbles. Thus, an inflammatory cytokine-targeting ultrasound contrast agent carrying IL-8 monoclonal antibody can provide unique advantages for researching the characteristics of atherosclerotic plaque.

## Introduction

Atherosclerosis is a common ischemic cardiovascular and cerebrovascular disease. Atherosclerotic progression eventually leads to the occurrence of severe cardiovascular events, such as myocardial infarction or stroke. As the prevalence of atherosclerosis increases globally due to an aging population, identifying atherosclerotic plaques will be important for early diagnosis and intervention, which will substantially decrease the healthcare burden.

Current non-invasive imaging modalities for atherosclerotic plaque detection include ultrasound, computed tomography (CT), magnetic resonance imaging (MRI), and angiography [17]. With the improvement of contrast-enhanced sonography techniques and the development of targeted contrast agents, microbubble contrast agents for ultrasound have emerged as a widely used tool that holds great potential in targeted contrast ultrasound imaging investigations [21].

Atherosclerosis occurs because of chronic injury to the endothelium, and angiogenesis is the most important pathological feature of atherosclerosis [12]. Ultrasound contrast microbubbles can clearly show the adventitia that nourishes atherosclerotic vessels as well as intraplaque neovascularization [4]. Previous studies of targeted imaging of atherosclerotic plaque have mainly focused on vascular endothelial growth factor receptor-2 [9], intercellular adhesion molecule-1(ICAM-1) [18, 19], vascular cell adhesion molecule-1 (VCAM-1), P-selectin [5], and oxidized low-density lipoprotein (OxLDL) [16]. While interleukin-8 (IL-8) is a key vasculogenesis regulator in atherosclerotic lesions and is capable of promoting angiogenesis and plaque formation, increasing evidence suggests that IL-8 is highly expressed within human atherosclerotic lesions ([2]; [14]).

Therefore, in the current study, acoustic contrast agents targeted to IL-8 were prepared and used for abdominal aortic plaque detection in a rabbit atherosclerosis model. We aimed to investigate the value of inflammatory factor-targeted, contrast-enhanced ultrasound (CEU) in atherosclerotic plaque visualization.

## Materials and methods

### Microbubble preparation

A solution of sulfur hexafluoride microbubbles (SonoVue, Bracco, Milan, Italy) was prepared with an average microbubble diameter of 2.5 μm and concentration of 17 mg/mL. Then, the microbubbles were conjugated to the antibodies by means of *N*-succinimidyl-3-(2-pyridyldithio)propionate (SPDP) conjugation. Briefly, mouse anti-human IL-8 monoclonal antibody (Bender MedSystems, Vienna, Austria) was dissolved in 0.01 mol/L PBS (pH 7.4) solution, followed by addition of a 20 mmol/L SPDP (Merck, Darmstadt, Germany) solution to a ratio of 1:15. The mixture was incubated for 60 min at 4 °C to react, and it then underwent five cycles of 8 min of centrifugation at 6,500 rpm followed by filtration through Microcon YM-30 filters (Millipore, Bedford, MA, USA) to remove the unbound SPDP.

Excess dithiothreitol (DTT, Invitrogen, Carlsbad, California, USA) dissolved in acetic acid was added to the solution of monoclonal antibodies containing pyridine disulfide groups (McAb-PDP) before incubation for 30 min at 4 °C. This mixture was then centrifuged for 8 min at 6,500 rpm and filtered three times through Microcon filters to remove unbound DTT, and the IL-8 monoclonal antibody solution with sulfhydryl (McAb-PDP-SH) was obtained.

The SonoVue microbubble suspension was reacted with 20 mmol/L SPDP solution at 4 °C for 60 min. The reaction solution was washed with 0.01 mol/L PBS (pH 7.4) using the floating method [8] to produce a microbubble suspension with pyridine disulfide groups. This suspension was immediately mixed with the McAb-PDP-SH solution and left to react at 4 °C overnight. The IL-8 monoclonal antibody-conjugated acoustic microbubble suspension was obtained after a final wash with PBS.

### Characterization of targeted microbubbles

#### Targeted SonoVue microbubble morphology

The IL-8 monoclonal antibody-conjugated SonoVue microbubble suspension and non-conjugated SonoVue microbubble suspension were smeared onto glass slides and observed under a microscope (Nikon Eclipse E200, Tokyo, Japan) to examine the structural properties, size, shape, and concentration of the microbubbles.

#### Slide agglutination test

The IL-8 monoclonal antibody-conjugated SonoVue microbubble suspension was mixed with either goat anti-mouse IgG serum (KPL Laboratories, Gaithersburg, MD, USA) or saline. The non-conjugated SonoVue microbubble suspension was mixed with either goat anti-mouse IgG or saline. These four mixtures were smeared onto clean slides and observed under a microscope to examine the morphology, structural properties, and other characteristics of the microbubbles [20].

#### Immunofluorescent staining

**Fig. 1:**
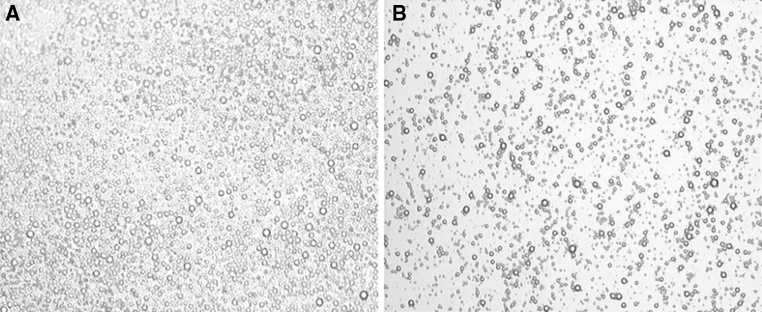
Light micrograph of SonoVue microbubbles. **a** Non-conjugated SonoVue microbubbles. **b** IL-8 monoclonal antibody-conjugated SonoVue microbubbles (×100)

Microbubbles conjugated with IL-8 antibody were added to PBS for oscillation, and the supernatant bubbles were decanted to another PBS solution for further shaking. This process was repeated 3 times. Impurities and non-conjugated IL-8 antibody remained at the bottom, while microbubbles floated to the surface. Thus the conjugated microbubbles were successfully separated. Samples of microbubbles were microscopically observed after each oscillation and washing to ensure that their nature (aggregation state of microbubbles, as in Fig. 1) was unchanged.

The non-conjugated microbubbles and IL-8-conjugated microbubbles were smeared on clean glass slides, and 1:100 FITC sheep anti-mouse IgG antibody (Merck, Darmstadt, Germany) was added following incubation at 4 °C for 60 min. Fluorescence microscopy was used to observe the fluorescent staining.

### Animal models and preparation

The animal experiments were approved by the institutional Animal Research Committee. Seventeen male Japanese white rabbits (1 month old) were used to establish a model of atherosclerosis of the abdominal aorta. Rabbits were fed a high-fat diet containing 1 % cholesterol, 7.5 % egg yolk powder, 8 % beef fat, and 83.5 % normal diet by weight, and they had free access to water. After 12 weeks of feeding, high-frequency ultrasound was performed to detect the formation of plaque within the abdominal aorta [15].

### In vivo targeted imaging of atheromatous abdominal aortas of rabbits

The auricular vein was cannulated, and the rabbits were anesthetized with intravenous sodium pentobarbital. Ultrasound imaging (Philips iE33 Ultrasound, Philips Medical Systems, Andover, MA, USA) of the aorta was performed with an L9-3 probe at a high-frequency of 7 MHz. The frame rate of CEU was 50 Hz with temporal resolution of 17.5 ms. The mechanical index was 0.08, and the dynamic range was 50 dB. All settings were kept constant during the experiment. Stable images of the abdominal aorta were obtained by scanning from the sub-xiphoid. CEU was started when atherosclerotic plaque was clearly seen with high-frequency ultrasound. CEU was first performed with the non-targeted SonoVue microbubble and then with the IL-8-targeted SonoVue microbubble 15 min later. The microbubbles (0.05 mL/kg) were quickly injected via the auricular vein, followed by infusion of 1 mL saline to flush the duct. Once contrast agent was injected, the CEU program was started to acquire continuous dynamic images for 3 min post-injection. Image data were analyzed offline using QLAB software (Philips Medical Systems, Bothell, WA, USA). A region of interest with the same size and location of the plaque was placed in the frame, and another area of 1.5 × 1.5 mm was placed in the adjacent arterial lumen and intima as the control region. Subsequently, a time-intensity curve (TIC) was plotted and used to calculate the characteristic parameters with the equation (t) = A*t* exp (−Alpha *t) + C, where A is the upslope rate, Alpha is the downslope rate, C is the initial time, and t is the initial time of ascending. The parameters measured with TICs were echo mean, peak intensity, wash-in time, time to peak, and time from peak to one-half.

### Histology and immunochemistry

The animals were humanely sacrificed after ultrasound imaging, and the abdominal aorta was excised for frozen section preparation and HE staining. The luminal surface was observed by microscopy (Nikon Eclipse E200, Tokyo, Japan).

To measure IL-8 expression in the plaques of the abdominal aorta, immunochemistry for IL-8 was performed. After deparaffinization and hydration, the paraffin sections were washed with tap water. After the addition of rabbit polyclonal antibody against IL-8 (Bioss, Beijing, China), the sections were incubated at room temperature for 60 min, washed with PBS, and stained using a MaxvisionTM2 HRP-Polymer anti-Mouse IHC Kit (Maxim Biotech Inc. Fuzhou, China). The sections were counterstained with hematoxylin and mounted.

To validate the neovascularization in the plaques of the abdominal aorta, immunohistochemistry was performed to detect CD34 expression. The plaques in the rabbit abdominal aortas were taken for paraffin sections. After dehydration, endogenous peroxidase was blocked, and the slides were pre-incubated with 1 % bovine serum albumin/PBS solution. Mouse monoclonal anti-CD34 antibody (Maxim Biotech Inc.) was added, the sections were incubated overnight at 4 °C, and they were stained using an immunohistochemical streptavidin peroxidase (SP) kit (Maxim Biotech Inc.). After color development with DAB chromogen, the sections were counterstained with hematoxylin and mounted for observation under a light microscope. The dense brown masses (CD34-positive staining) indicated a dense microvessel.

### Statistical analysis

All experimental data are reported as the mean ± SD and were analyzed using SPSS 13.0 (SPSS Inc, Chicago, IL, USA). A paired *t* test was performed to compare the differences between the control and targeted imaging groups. Findings with *P* < 0.05 were considered statistically significant.

## Results

### Morphology of microbubble conjugation

The concentration of the microbubble suspension was 17 mg/ml. The non-conjugated microbubbles had a uniform size with a diameter of 2–4 μm, and the distribution was also uniform with a single and scattered pattern (Fig. 1a). Whereas the IL-8 monoclonal antibody-conjugated SonoVue microbubbles had relatively low densities, the size, shape and structural properties of the microbubbles were similar to those of non-conjugated microbubbles, as they had a uniform size and distribution (Fig. 1b).

### Slide agglutination test

**Fig. 2:**
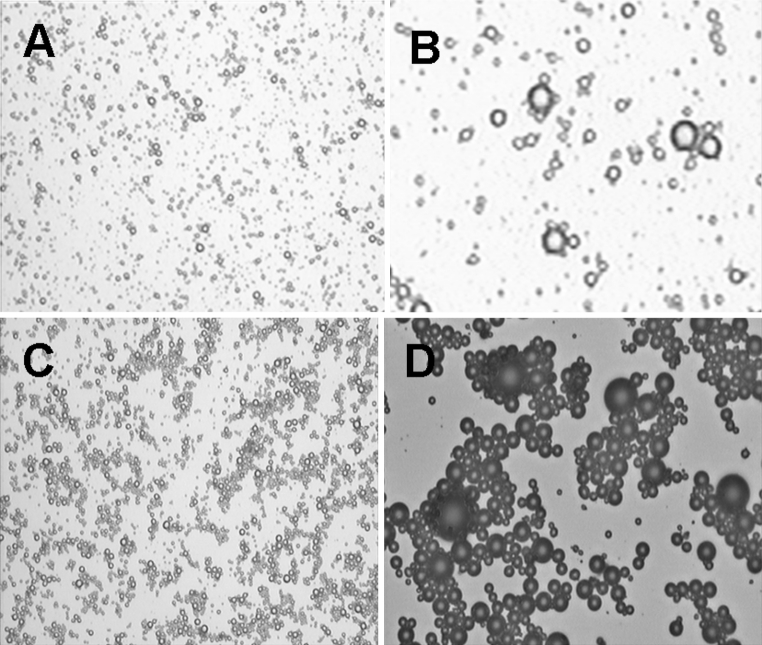
Light micrograph of the mixture of SonoVue microbubbles and goat anti-mouse serum. **a** Non-conjugated microbubbles (×100). **b** Non-conjugated microbubbles (×400). **c** IL-8 monoclonal antibody-conjugated microbubbles (×100). **d** IL-8 monoclonal antibody-conjugated microbubbles (×400)

The non-conjugated SonoVue microbubbles did not present agglutination when mixed with goat anti-mouse serum, as their size and scattering were uniform (Fig. 2a). Two types of microbubbles did not show any obvious changes in bubble size, shape or macrostructural properties in the presence of normal saline, again indicating no agglutination. When goat anti-mouse serum was added, the IL-8 monoclonal antibody-conjugated SonoVue microbubbles agglutinated readily, with several microbubbles gathered in string-like clusters but no longer scatted (Fig. 2b, c), indicating that IL-8 monoclonal antibodies had been successfully conjugated to the shell surface of the microbubbles.

### Immunofluorescent staining of the microbubbles

**Fig. 3:**
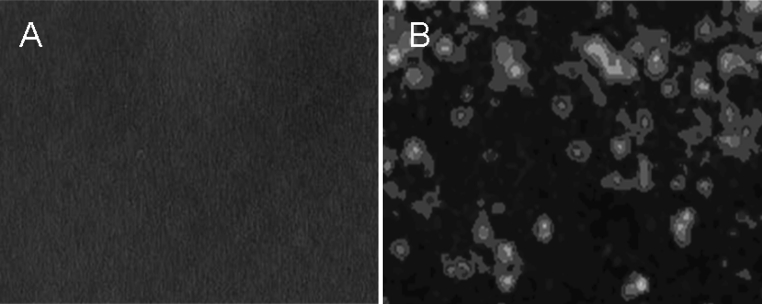
Immunofluorescence image of IgG-FITC-stained SonoVue microbubbles. **a** No fluorescence was observed on the surface of non-conjugated SonoVue microbubbles. **b** *Bright-green* fluorescence can be observed on the surface of IL-8-conjugated SonoVue microbubbles (×400). (Color figure online)

When mixed with sheep anti-mouse–FITC, no fluorescence was observed on the surface of uncoupled SonoVue microbubbles with a fluorescence microscope, whereas there was obvious green fluorescence from IL-8 antibody-conjugated bubbles (Fig. 3).

### In vivo ultrasound imaging of plaques

**Fig. 4:**
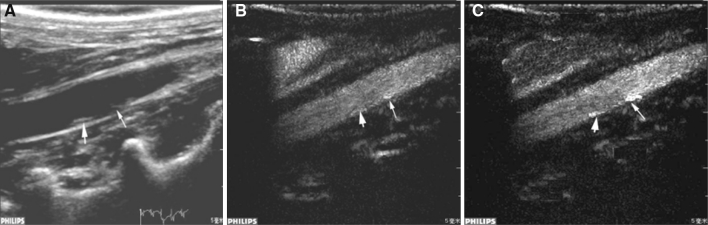
Representative ultrasound images of the atherosclerotic plaques in the rabbit abdominal aorta. **a** Two-dimensional image showing a plaque (*large arrow*); a soft plaque was invisible (*small arrow*). **b** Contrast ultrasound imaging with normal microbubbles showing two plaques with stronger echo compared with the two-dimensional view. The enhancement of the echo was more apparent for the soft plaque (*small arrow*). **c** Contrast ultrasound imaging with IL-8-targeted microbubbles showing two plaques with a markedly stronger echo compared with normal contrast ultrasound

After high-fat diet feeding, 19 plaques were detected using two-dimensional ultrasound examination in 17 rabbits, with soft plaques dominating. Plaques were visualized as hypoechoic in six lesions, isoechoic in seven lesions, and hyperechoic in six lesions. Thirteen plaques occurred at the abdominal aortic bifurcation, and six were located at other sites. CEU with normal SonoVue microbubbles identified an additional three plaques. With IL-8-targeted microbubbles, CEU detected two new hypoechoic plaques that were missed by normal CEU (Fig. 4).

During the contrast ultrasound imaging of abdominal aortas with normal or IL-8-targeted microbubbles, contrast was initially observed in the vessel lumen, and then it gradually filled the adventitia and the whole plaque. The vasa vasorum was strongly contrasted as well. Generally, the contrast could be visualized in the lumen 3–6 s after the injection of contrast agent, in the intima at 4–8 s, and in the atherosclerotic plaque at 5–10 s.

**Fig. 5:**
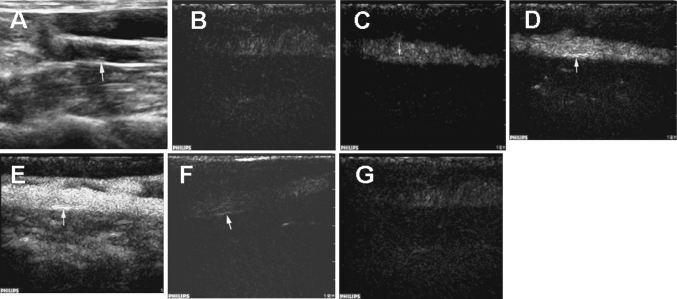
Sequential ultrasound images of IL-8-targeted imaging for an atherosclerotic plaque of the rabbit abdominal aorta. **a** Conventional two-dimensional view. **b**–**g** Sequential targeted CEU view: **b** 3.8 s post-contrast agent injection, **c**: 4.3 s post-contrast agent injection, **d** 4.9 s post-contrast agent injection; **e** 9.8 s post-contrast agent injection, **f** 68 s post-contrast agent injection, **g** 176 s post-contrast agent injection

However, contrast imaging with IL-8-targeted microbubbles provided enhanced echo intensity of atherosclerotic plaques compared to the normal contrast imaging. Detailed information of the surrounding vasa vasorum was also clearer. Briefly, the contrast gradually increased from the intima and wall of the lumen to plaque in a sparse and spot-like pattern, and it connected with the capillary vessels that originated from the intima on the edge of the plaque lesions. These signs were even more pronounced for hypoechoic plaques (Fig. 5).

### Time-intensity analysis

**Fig. 6:**
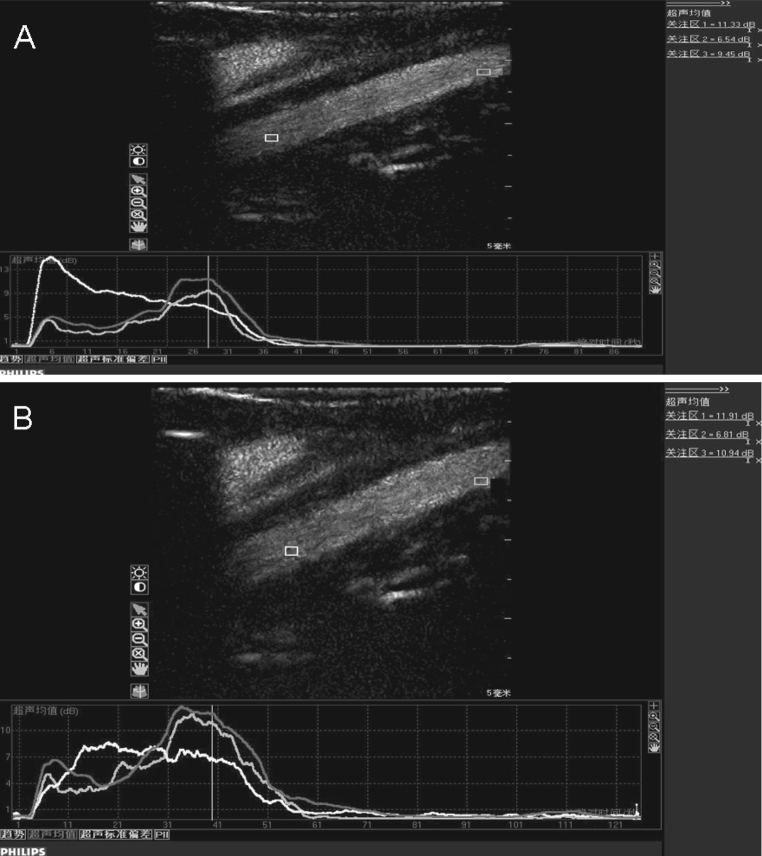
The time-intensity curves of contrast ultrasound imaging with normal microbubbles (**a**) and IL-8-targeted microbubbles (**b**). The *yellow*, *blue* and *red* curves indicate the intensity of the lumen, intima and plaques, respectively. (Color figure online)

The time-intensity curves based on the normal and IL-8-targeted ultrasound imaging presented similar trends for the contrast of the lumen, intima and atherosclerotic plaques. The peak intensity was followed by a fast ascending phase after injection of the contrast agents, and a slow descending phase occurred after the peak intensity pulse (Fig. 6). The contrast enhancement was observed in the lumen 3–6 s after injection, 4–8 s in the intima with a gradually higher echo, and 5–10 s in the atherosclerotic plaque.

The time-intensity data using the targeted and the non-targeted contrast agent are compared in Table 1. In the lumen, there were no significant difference in wash-in time, time to peak or time from peak to one-half between contrast ultrasound imaging with normal microbubbles and IL-8-targeted microbubbles. The comparison also presented no significant difference in intraluminal echo mean or peak intensity. The common contrast characteristics of IL-8-targeted microbubbles and non-targeted microbubbles in the lumen indicated that the prepared IL-8-targeted SonoVue microbubbles are physically stable.

**Table 1:** Comparison of time-intensity curves of targeted and non-targeted contrast ultrasound imaging

		Echo mean (dB)	Peak intensity (dB)	Wash-in time (s)	Time to peak (s)	Time from peak to one-half (s)
Non-targeted UCA (*N* = 22)	Lumen	4.4 ± 1.9	12.3 ± 4.9	3.5 ± 2.3	15.3 ± 3.9	29.7 ± 6.3
	Intima	3.5 ± 1.6	12.7 ± 5.6	6.1 ± 2.2	18.2 ± 4.0	35.0 ± 5.9
	Intima/lumen ratio	0.79 ± 0.21	1.01 ± 0.23	1.4 ± 0.55	1.15 ± 0.45	1.18 ± 0.48
	Plaque	4.2 ± 2.1	15.4 ± 5.4	7.2 ± 2.4	18.5 ± 4.3	38.5 ± 5.6
	Plaque/lumen ratio	0.95 ± 0.23	1.23 ± 0.34	1.98 ± 0.55	1.2 ± 0.53	1.29 ± 0.49
Targeted UCA (*N* = 24)	Lumen	4.6 ± 2.5	12.5 ± 6.7	3.7 ± 2.9	15.9 ± 4.9	30.2 ± 6.8
	Intima	5.5 ± 1.6*	16.3 ± 7.2*	5.5 ± 2.6	18.2 ± 5.2	43.8 ± 7.8*
	Intima/lumen ratio	1.20 ± 0.28*	1.31 ± 0.33*	1.4 ± 0.48	1.14 ± 0.51	1.41 ± 0.53*
	Plaque	6.8 ± 1.8*	18.7 ± 5.9*	6.9 ± 2.8	20.5 ± 5.6	46.7 ± 7.9*
	Plaque/lumen ratio	1.47 ± 0.36*	1.50 ± 0.37*	1.9 ± 0.49	1.2 ± 0.57	1.53 ± 0.47*

*UCA* ultrasound contrast agent

*Significant difference between targeted and non-targeted UCA (*P* < 0.05). Values are provided as the mean ± SD

For the intima and the plaque, the echo mean and peak intensity in targeted imaging were significantly increased compared with normal CEU (*P* < 0.05), indicating that IL-8-targeted SonoVue microbubbles can enhance the echoes of the intima and plaque. In addition, plaques under targeted imaging were easier to identify because both the intima/lumen ratio and plaque/lumen ratio were increased compared to normal CEU (*P* < 0.05). Wash-in time and time to peak demonstrated no difference between conditions, suggesting that the mechanism by which contrast improved in the intima and plaques is the same for the two contrast agents. The time from peak to one-half was significantly prolonged with targeted imaging (*P* < 0.05), revealing an extended combination of contrast agent with intima and plaque, which facilitated the detection of plaques.

### Histology and immunochemistry

**Fig. 7:**
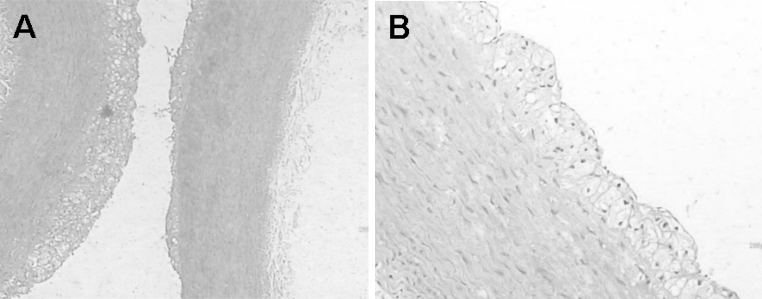
Typical histological images of rabbit abdominal aortic atherosclerotic plaque (**a**×100, **b**×400)

All atherosclerotic plaques of the abdominal aorta detected with contrast imaging were confirmed with histological examination. Fig. 7 shows histological images of atherosclerotic plaque specimens. The intima was markedly thickened. The atherosclerotic plaques consisted of large numbers of foam cells, which were widely distributed in the media and subintimal region. Smooth muscle cell proliferation, with a disorganized arrangement, was observed in the media layer.

**Fig. 8:**
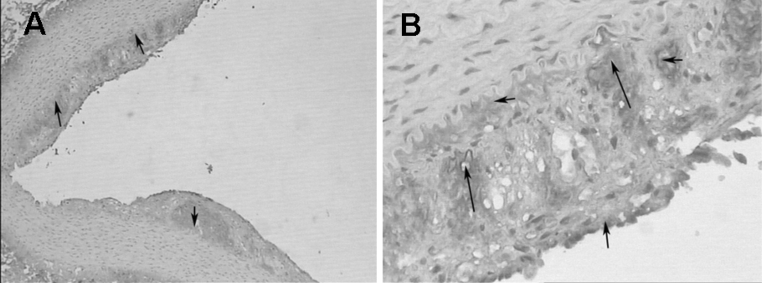
Immunohistochemical expression of IL-8 in atherosclerotic plaques in the rabbit abdominal aorta. *Brown*-stained area indicates positive IL-8 expression. (**a** ×40; **b** ×200). (Color figure online)

Immunohistochemical examination of IL-8 expression in atherosclerotic plaques showed a mass of brown (positive) substance at the basal and inner parts of the atherosclerotic plaques, indicating the high expression of IL-8 in rabbit abdominal aortas (Fig. 8).

**Fig. 9:**
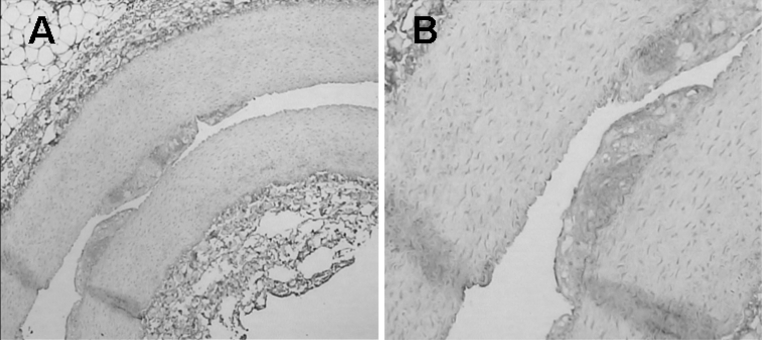
The missed soft plaque in two-dimensional images was identified by CD34 immunohistochemistry by its greatly increased *brown* staining (CD34-positive) in the inner part and base of the plaque compared with adjacent healthy intima. This result indicates a high neovascular density for the plaque, which is consistent with enrichment of the targeted microbubbles in contrast imaging. (**a** ×40; **b** ×100). (Color figure online)

Finally, we investigated neovascularization in the atherosclerotic plaques in the rabbit abdominal aorta. The plaques identified by contrast imaging showed strong brown staining (CD34) in the inner part and base of the plaque compared with adjacent healthy intima (Fig. 9). This indicates a high neovascular density in the plaques, which is consistent with enrichment of the targeted microbubbles in contrast imaging.

## Discussion

Currently, ultrasound contrast imaging is of intense interest in the field of atherosclerotic plaque detection. Acoustic microbubbles are a type of intravascular contrast agent that can be conjugated with targeted ligands to bind to lesions with high specificity and high affinity [6, 18, 19]. In this study, an IL-8 monoclonal antibody was conjugated to the liposome contrast agent SonoVue via an active targeting approach, and the prepared IL-8-targeted contrast agent was successfully applied to ultrasound imaging of rabbit abdominal aorta atherosclerotic plaque and showed increased detection sensitivity compared with non-targeted SonoVue microbubbles.

The development of targeted ultrasound contrast agents is still in the nascent phase. Several studies have shown that covalent coupling is an ideal method to conjugate specific antibodies to the surface of the acoustic microbubble, and this method has been successfully used to develop antibody-conjugated liposome microbubbles [3, 20]. SonoVue is a widely used blood pool ultrasound contrast agent. In the present study, IL-8 monoclonal antibody was covalently linked to the liposome shell of the SonoVue microbubble via a disulfide bond using the SPDP method, and the size, shape, and macrostructural properties of the microbubbles were stable. This result indicates that the physical and biological characteristics of the microbubbles can be kept constant after SPDP cross-linking; therefore, the IL-8-conjugated SonoVue microbubbles can be safely applied in vivo. In addition, goat anti-mouse IgG serum agglutinates with mouse anti-human IL-8 monoclonal antibody if the primary antibody (in this case the IL-8 monoclonal antibody) exists in the microbubble compound. Our slide agglutination test revealed extensive agglutination when the IL-8 monoclonal antibody-targeted acoustic microbubble suspension was mixed with goat anti-mouse IgG. Immunofluorescent staining showed that only IL-8 antibody-conjugated microbubbles presented fluorescence when mixed with sheep anti-mouse IgG-FITC. Both of these results indicate that an IL-8 monoclonal antibody has been successfully coupled to the shell surface of the microbubbles. These characterizations of the targeted microbubbles demonstrate that the specific contrast agent, namely IL-8-conjugated SonoVue microbubbles, can be successfully developed via the SPDP method.

Due to the increasing trend of cardiovascular and cerebrovascular events in many patient populations, the identification and analysis of atherosclerotic plaque are of utmost importance for the reduction of these severe events. Neovascularization-relevant inflammation, as well as the inflammatory factors involved in this process, are considered indicators of the stability of the atherosclerotic plaque and are therefore the focus of intense investigation [10].

IL-8 is the representative factor of the chemokine CXC group. It is mainly secreted by monocyte-macrophages at the lesion site, with high expression at sites of human atherosclerotic plaque [7]. It is the main chemokine that continually induces immune cells to invade atherosclerotic plaques. IL-8 is an important angiogenic factor at plaque lesion sites, promoting the formation and progression of atherosclerotic plaques. It is worth noting that IL-8 is one of the inflammatory factors secreted by macrophages, monocytes and endothelial cells, suggesting that angiogenesis is associated with inflammatory reactions [13].

In this study, we initially applied an IL-8 monoclonal antibody-targeted acoustic contrast agent to access the atherosclerotic plaques in the rabbit abdominal aorta. We found that the contrast curves for the lumen, intima and plaque in the presence of targeted and non-targeted contrast agents shared similar trends. Moreover, targeted CEU with IL-8 antibody-conjugated microbubbles enabled us to observe the blood-supply characteristics of atherosclerotic plaques in more detail, i.e. the feeding vessels of the plaques. These blood-supply features are closely related to the stability of the plaque [11, 22]. Because new intraplaque vessels primarily originate from preexisting capillaries in the vascular wall by budding or non-budding mechanisms, the periphery of the plaque usually presents with denser capillaries and inflammatory cells and more severe inflammatory reactions, Hence, it is easier to induce angiogenesis at the plaque periphery than in central regions. As a result, the aforementioned contrast characteristics will be more prevalent for unstable plaques. The relationship between the plaque stability and the targeted CEU features will be further investigated along with pathological features in our future studies.

A limitation of ultrasound is that B-mode imaging is a qualitative and semi-quantitative technique, which is mainly used to subjectively observe the perfusion trend in the plaques during contrast imaging. In our study, B-mode imaging provided a preliminary basis for the quantitative analysis with QLAB software before further observation of the targeted microbubble binding.

In summary, a novel ultrasound contrast agent, IL-8-targeted SonoVue microbubbles, was successfully developed and used to detect atherosclerotic plaques in the rabbit abdominal aorta. CEU with the agent can effectively identify atherosclerotic plaques in vivo, including those plaques not detected with non-targeted CEU imaging. This method may become a valuable imaging technique for diagnosing atherosclerotic lesions in clinical practice.
